# Reply to: A large mid-Holocene estuary was not present in the lower River Murray, Australia

**DOI:** 10.1038/s41598-021-89076-9

**Published:** 2021-06-08

**Authors:** T. C. T. Hubble, A. M. Helfensdorfer, T. A. Job, H. E. Power

**Affiliations:** 1 School of Geosciences, The University of Sydney, Sydney, NSW 2006 Australia; 2 School of Environmental and Life Sciences, The University of Newcastle, Callaghan, NSW 2308 Australia

**Keywords:** Limnology, Hydrology

**replying** **to**: J. Tibby et al.; *Scientific Reports* 10.1038/s41598-021-90025-9 (2021).

## Introduction

Three substantive criticisms^1^ have made been of our studies^2,3^ which identified an extensive central basin environment that we posit occupied the entire one-to-three- kilometre width of the Murray Gorge for at least 140 km of its length due to raised water levels generated by the mid-Holocene sea-level highstand. Specifically, these criticisms challenge our two key findings that: (1) the identified central basin acted as a sediment-trap between 8.5 and 5 cal kyr BP likely interrupting and modifying the amount of fine-grained fluvial sediment delivered to the ocean such that; (2) conclusions about Murray-Darling River flows during the mid-Holocene based on interpretation of the sediment record presented in two deep-water cores recovered offshore the Murray River’s mouth^4^ should be re-evaluated. Here, we refute Tibby et al's three criticisms^1^.

Criticism 1 challenges our identification of probable brackish-fresh and fresh-brackish waters in the Murray Gorge during the Holocene sea-level highstand. This objection^1^ is based on the distribution of Indigenous midden and burial sites located adjacent to, but not within, the present-day floodplain^5,6^. The middens have been dated on the basis of several charcoal fragments and abundant remains of the mussel *Velesunio ambiguus,* a generally freshwater species that can tolerate salinities up to 3 g L^−1^ ^9^. On this basis it is suggested^1^ that the “continuity of human occupation and reliance on freshwater from c. 8400 yr BP to the time of European occupation” indicates a large estuary could not have existed.

In our studies^2,3^, we used hydrological models as a tool to understand processes, not to exactly replicate reality. Our modelled salinity values are indicative of a range of possible conditions extant at the mid-Holocene highstand, not actual, absolute values apparently assumed by the critique. Nevertheless, the central basin of an estuarine system is defined by geomorphology not salinity and this zone can present salinities that range from marine to fresh. Salinities up to 1.2 g L^−1^ are currently considered palatable for human consumption^10^, while salinities between 3 g L^−1^ and 6 g L^−1^ are considered safe for watering beef cattle^10^. The range of possible Holocene highstand salinities we modelled in the vicinity of the midden sites are at the fresher end of this spectrum, from fresh-brackish (0.2 g L^−1^ to 0.9 g L^−1^) to brackish-fresh (0.9 g L^−1^ to 1.8 g L^−1^; Fig. 3^2^, Fig. 2)^3^ and are well below the ~ 3 g L^−1^ salinity tolerance of *V. ambiguus*^9^.

The salinity zones indicated by our models are therefore entirely consistent with the presence of *V. ambiguus* and unlikely to have been an impediment to continuous human occupation of the lower Murray valley after 8.4 cal kyr BP as posited^1^. We note that: a) Australia’s first nations peoples thrived in locations that presented much more challenging conditions than those that we modelled for the lower Murray; b) the majority of the dated shell materials are younger than 6.0 cal kyr BP, when our models indicate that conditions were fresher due to the lower sea level (Fig. 4a^2^, Fig. 2^3^); and c) the presence of authigenic pyrite^11,12^ is indicative of a source of marine sulphate^13^ in 6 to 6.5 cal kyr BP laminated muds sampled elsewhere at Monteith^11^ and 50 km further upstream^11^.

Further, sapropelic units derived from the accumulation of freshwater algae (including the described *Coorongite*^1^) that underly siliciclastic mud deposits in the Lower Lakes region were likely deposited in freshwater lakes not connected to the primary fluvio-estuarine system during the mid-Holocene^7^ and therefore cannot be used to assert freshwater conditions in the estuary^1^. Finally, the assertion that “sub-fossil diatoms in the Lower Lakes also indicate the dominance of freshwater at this time [6,930 ± 150 yr BP]”^1^ contradicts evidence of brackish to salty conditions for Lake Albert between 7.9 and 6.0 cal kyr BP^8^.

Criticism 2^1^, disputes the elevation of sea level that was selected to represent the mid-Holocene highstand in our modelling. This issue has been raised^14^ and refuted^15^ elsewhere. While the critique^1^ accepts a possible local mid-Holocene highstand of + 1 m, it categorically rejects a + 2 m highstand. The critique presents sea-level data for South Australia^1^ to demonstrate sea-level variation in the area after 12 cal kyr BP to support an assertion that “local sea level was > 20 m lower than at present^16^” at time of hypothesised estuary commencement [ca. 8.5 cal kyr BP]. Two separate arguments justify our selection of a + 2 m sea level for our modelling^2,3^.

**Figure 1:**
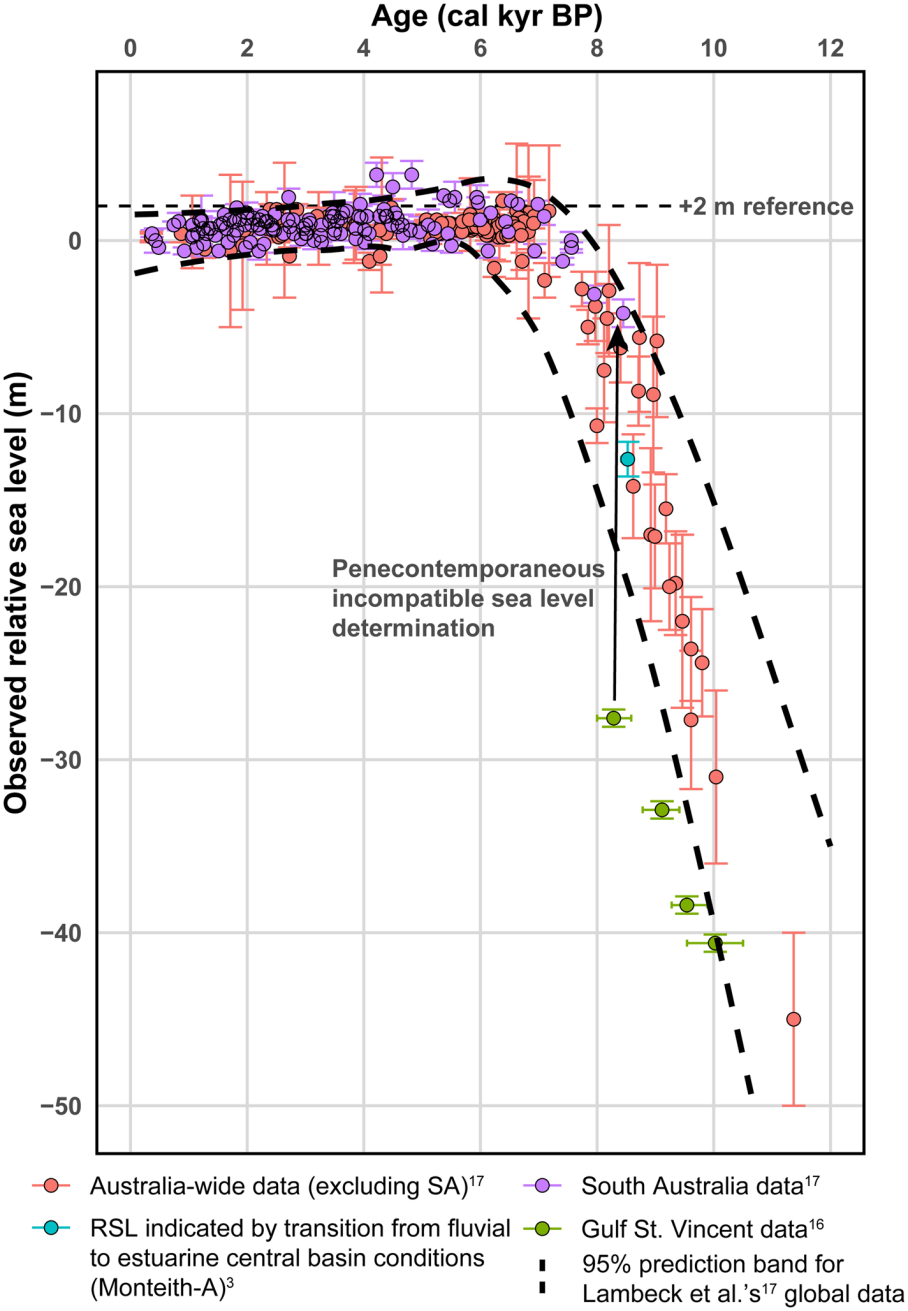
Observed relative sea levels during the Holocene. Australia-wide (excluding South Australia; n = 182), South Australia (n = 129), and global (n = 968) data are sourced from Lambeck et al.^17^. Sea level data from Gulf St. Vincent (n = 4; intertidal indicators only)^16^ were calibrated using the Marine20 radiocarbon age calibration curve^20^ using a ΔR of 84 yr and a standard deviation of 59 yr (as performed by Tibby et al.^1^) in CALIB 8.1^21^. 2σ age uncertainties for the Gulf St. Vincent data are included as horizontal error bars. The modelled 2σ age uncertainty (197 yr) for the Monteith-A transition is obscured by the plotted point. Lambeck et al.^17^ statistically incorporated age uncertainties into the elevation uncertainties which are included here. Upper and lower 95% prediction limits for global Holocene RSL data (plotted as black dashed lines) were calculated through locally weighted regression smoothing (LOESS) using *R 4.0.4*^24^ and the *msir* (v1.3.3^25^) package. The Gulf St. Vincent determinations of sea level between -28 m to -33 m between 8 cal kyr BP and 9 cal kyr BP are incompatible with the penecontemporaneous South Australian determinations of sea level between −2 m and −4 m at 8–8.4 cal kyr BP (see arrow). The older and deeper observations differ significantly from global observations and were excluded from the South Australian dataset by Lambeck et al.^17^ as “observations providing only limiting estimates”.

Firstly, we present and compare the South Australian data set used in the critique^1^ to high-quality global data sets^17^ which include Australian data derived from corals and sedimentary records of the east, north, and western Australian coasts (Fig. 1). It is evident that the progression of mid-Holocene sea-level rise is poorly constrained by the South Australian data^16^ as they present two strongly contradictory estimates of the sea level for the period between 9 and 7.5 cal kyr BP.

**Figure 2:**
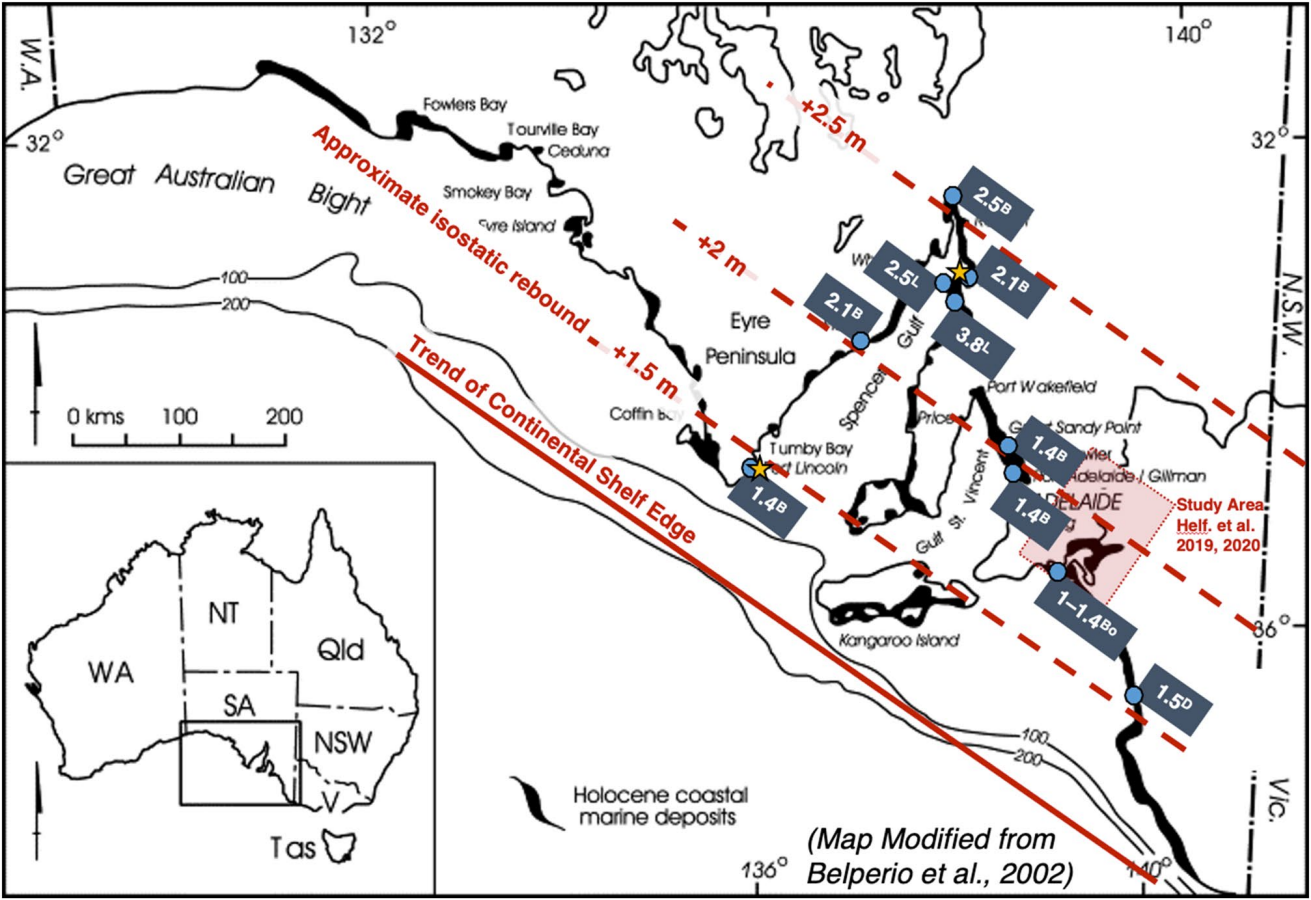
Map of coastal South Australia showing the distribution of indicators for Holocene highstand sea-level elevations presented by Belperio et al.^16^ (B), Lambeck et al.^17^ (L), Dillenburg et al.^22^ (D) and Bourman et al.^23^ (Bo). Here we present the maximum indicated relative sea-level elevation (in m) at each site (intertidal indicators; blue circles and associated labels) as well as approximated isostatic rebound contours (dashed red lines) oriented parallel to the approximate trend of the continental shelf edge (solid red line). These contours are representative values of post-mid-Holocene continental-edge load-stress relaxation induced^17^ uplift, i.e., the systematic increase of hydro-isostatic uplift with distance from the shelf margin identified for the area by Belperio et al.^16^. We note that mid-Holocene highstand relative sea levels are poorly constrained between Port Lincoln and Port Pirie (gold stars; modelled as + 1 and + 2 m respectively in Belperio et al.^16^), with sea level indicators proximal to the contour that passes through the mid-point of our study’s modelled spatial domain ranging from 1.4 to 2.1 m. As sea level estimates within this region are variable, we assert that our choice of + 2 m as an end-member value for modelling the mid-Holocene highstand mean sea-level is reasonable given the available data.

Offshore estimates^16^ from Gulf St. Vincent suggest that relative sea level rose from about -33 m to about -28 m between 9 and 8.3 cal kyr BP (Fig. 1) while onshore estimates locate the sea surface at -4 m at 8.4 cal kyr BP, rising to present day levels by around 7.0 cal kyr BP (Fig. 1 here; and Fig. 2)^1^. The shallower and younger data^16^ are in close agreement with global and other Australian data but the older and deeper values are both discrepant with, and significantly lower than, sea level values recorded by the global and other Australian datasets. The transition from lower sea levels (> 30 m below present) at 10 cal kyr BP to near-modern sea levels (circa 5 m below present) at 8 cal kyr BP is evident in both data sets and is contemporaneous with our identified transition from fluvial to central basin conditions and initiation of a backwater environment at Monteith (−12.6 m at 8.5 cal kyr BP, Fig. 1). The fluvial to estuarine transition we identify is therefore entirely concordant with the available regional and global sea level data.

Secondly, we recognise that glacio-hydro-isostatic adjustment of the South Australian coastline resulted in spatially variable highstand sea levels, with uplift observed to progressively increase with distance from the continental shelf^16^. Figure 2 presents a map of the South Australian coast showing the position of the continental shelf-edge and a modelled set of contours for hydro-isostatic rebound. These contours are oriented parallel to the shelf-edge whose positions comply with the accepted regional increase^16^ of post-highstand uplift with increasing distance from the shelf-edge. Sea level estimates across the modelled spatial domain are variable, but generally confirm that a Holocene highstand sea-level value of + 2 m at Murray Mouth is a reasonable estimate and ‘fit for purpose’ for our modelling studies which applied end-member conditions to investigate the possible range of conditions and driving processes.

Criticism 3 describes the modelling as “seriously flawed” due to three factors: (1) an “unjustifiable sea level highstand (+ 2 m), and width of the Murray Mouth in several scenarios in the model setup”^1^, which is addressed above or elsewhere^15^; (2) the use of the salinities during the Millennium Drought as the initial condition for the model; and (3) the model run durations.

The objection^1^ to the use of the salinities during the Millennium Drought as the initial condition for the model is based on an untested assumption that the lower Murray River was fresh during the Holocene highstand. However, as described above, there is clear evidence to the contrary, hence the use of salinities during the Millennium drought is an equally valid starting point for the modelling. In the modelling we conducted^2,3^, initial salinity values were applied at each cell based on salinity data taken from 25 gauging stations throughout the region at the peak of the Millennium drought. This was deemed appropriate as the barrages are in place to curtail saline intrusion and therefore regional salinities are held fresher than would naturally occur without the presence of barrages.

The ‘model run duration’ objection is addressed through the use of the initial condition dataset described above which reduced the duration of the spin-up phase required, allowing meaningful results to be obtained in a significantly shorter run time. Adopting this initial condition dataset, drought (D_-_) and pre-regulation average (D_av_) scenarios were run for 20 days while flood (D_+﻿_) scenarios were run for 31 days which included a six-day peak discharge flow period and three-day transition periods between peak and normal flow rates. These durations were sufficient for models to run beyond the spin-up phase and reach steady state, as confirmed by reviews of hydrograph phasing extracted from model outputs. Water levels throughout the model rose to the flood peak and subsequently returned to pre-flood water heights well within the 31-day model scenario, clearly demonstrating that the simulation run time is sufficient to resolve the problem at the intended scale and resolution.

Further, we reiterate that the modelling presented in our studies was intended to constrain possible end-member values of variables to identify the processes most important for driving water levels, flow velocities, and sediment deposition in the lower Murray River and Lower Lakes during the mid-Holocene^3^. The modelling was not intended to replicate a “true” scenario and was never presented as such. Rather, our models represent end-members of the range of plausible possibilities. Our models do, however, clearly demonstrate that a + 2 m Holocene highstand results in the formation of a valley-wide, low-flow velocity environment that is > 100 km in length with salinities higher than at present day sea levels. Thus, our modelling clearly demonstrates that it is the Holocene sea-level highstand that was the primary factor responsible for generating a large, mid-Holocene estuary and central basin within the lower Murray Gorge resulting in the deposition of the > 100 km long, valley-wide, > 10 m thick, so-called ‘Mannum Mud’ deposit^3,18,19^.
